# Case Report: Pediatric Erdheim–Chester disease with cerebral ventricular system infiltration and epiphyseal involvement

**DOI:** 10.3389/fped.2026.1903598

**Published:** 2026-08-25

**Authors:** Jie Yan, Ju Li, Qian Li, Xiao He, Ming Zhu, Luping Fan, Ronghui Li, Yan Long, Shuixian Huang

**Affiliations:** 1 Department of Radiology, Gejiu People’s Hospital, Gejiu, Yunnan, China; 2 Department of Pathology, Gejiu People’s Hospital, Gejiu, Yunnan, China

**Keywords:** case report, central nervous system, Erdheim–Chester disease, histiocytosis, magnetic resonance imaging

## Abstract

**Background:**

Erdheim–Chester disease (ECD) is an extremely rare non-Langerhans cell histiocytosis, a category of hematologic disorders characterized by the proliferation of foamy histiocytes. ECD predominantly affects adults, and pediatric cases with cerebral ventricular system involvement are exceptionally rare, with only a few previous reports. Moreover, epiphyseal involvement has not been characterized by magnetic resonance imaging (MRI) in children with ECD. Here we present a pediatric case of ECD involving both the cerebral ventricular system and epiphyses, highlighting diagnostic challenges and novel imaging findings relevant to pediatric hematologists.

**Case presentation:**

An 11-year-old boy presented with progressive gait disturbance, growth retardation, left knee pain, dysuria, and nocturia over two years. Imaging revealed a mass in the right lateral ventricle, patchy osteosclerosis of the tibial epiphyses, and bilateral cerebellar white matter lesions showing the “crab sign” (central cavitation within the dentate nuclei on Fluid Attenuated Inversion Recovery (FLAIR) sequences). The ventricular mass was surgically resected. Histopathology and immunohistochemistry (CD68+, CD163+, CD1a−) confirmed the diagnosis of ECD, and molecular analysis detected a BRAF V600E mutation (6.40%). The patient received dabrafenib (a BRAF inhibitor), interferon alpha-2b, and desmopressin. At the 32-month follow-up, the patient had become non-ambulatory and exhibited speech delay, partly due to poor medication adherence, highlighting that even brief interruption of dabrafenib therapy can lead to rapid disease progression. For pediatric hematologists, this case strongly demonstrates that continuous administration of dabrafenib is essential for controlling disease in children with BRAF V600E-mutated ECD.

**Conclusions:**

This report presents a rare pediatric case of ECD characterized by involvement of both the cerebral ventricular system and epiphyses. We suggest that persistent bladder wall thickening (≥1.5 cm) may serve as a novel imaging indicator of urinary tract involvement in ECD, although histological confirmation is required. The “crab sign” could aid in the early diagnosis of central nervous system involvement. Importantly, this case illustrates that continuous administration of dabrafenib is crucial for disease management in children with BRAF V600E-mutated ECD, as even a brief interruption in therapy can result in rapid and severe deterioration. Our findings highlight the necessity of multimodal imaging and molecular analysis for accurate diagnosis and targeted therapeutic strategies in pediatric ECD.

## Background

ECD is a rare, nonhereditary, non-Langerhans cell histiocytosis first described by Chester and Erdheim in 1930 (1). It was formally recognized as a neoplasm in the WHO Classification of Tumours of Haematopoietic and Lymphoid Tissues in 2016 (2). The disease predominantly affects middle-aged and elderly individuals (mean age at onset 53 years) with a male predominance (3, 4). Pediatric ECD is extremely rare, with fewer than 50 cases documented in the literature (5). To date, only two cases of ECD presenting as a ventricular system mass have been reported (4, 6).

Despite the growing recognition of ECD as a neoplastic disorder, pediatric cases remain exceedingly rare, and the clinical and imaging spectrum in children is poorly characterized. In particular, cerebral ventricular system involvement has been documented in only a few pediatric cases, and the MRI features of epiphyseal involvement have not been described in the pediatric ECD literature. Furthermore, the optimal management strategy for pediatric ECD with central nervous system involvement, especially regarding BRAF inhibitor therapy, remains incompletely defined. Here we present a pediatric case of ECD with concurrent cerebral ventricular system infiltration and epiphyseal involvement, aiming to: (1) expand the imaging phenotype of pediatric ECD by describing novel MRI findings; (2) highlight the diagnostic challenges posed by atypical presentations in children; and (3) provide clinical evidence supporting the importance of continuous dabrafenib therapy in BRAF V600E-mutated pediatric ECD.

## Case presentation

### Patient information

An 11-year-old boy was admitted to our hospital with a persistent two-year history of unsteady gait. His parents reported that his height had not increased over the preceding four years, making him significantly shorter than his peers of the same age and sex. During the last two years, he had difficulty walking on uneven surfaces (e.g., stairs or slopes), with abduction and swaying of the lower limbs, accompanied by marked left knee pain. Additionally, he complained of painful micturition and increased nocturnal frequency during the month prior to admission. Physical examination revealed the following: height, 115 cm; weight, 21 kg; delayed growth; facial rash; scattered red rashes on the abdomen; and no acne or pigmentation. The extremities exhibited normal muscle strength and tone. The bilateral Babinski and Chaddock signs were positive. The remainder of the neurological examination was unremarkable.

The patient's medical history was unremarkable for any chronic illnesses, prior hospitalizations, or significant infections. Regarding family history, a detailed three-generation pedigree was obtained through interview with the parents. All family members (including parents, grandparents, and siblings) had no history of histiocytic disorders, hematological malignancies, or other similar conditions, nor any known hereditary diseases. The parents were non-consanguineous, and there was no family history of short stature, orthopedic disorders, or neurological diseases. Psychosocial history revealed that the patient lived with his parents in a rural area of Yunnan Province, China, with limited access to specialized medical care prior to diagnosis. He was a student with average academic performance before symptom onset; however, progressive gait impairment had led to school absenteeism and social isolation over the two years preceding diagnosis.

Electroencephalography: showed recurrent sharp-wave epileptiform discharges in the frontal and frontal midline regions.

### Laboratory findings

Tumor markers: including CA19-9, CA-125, alpha-fetoprotein, and carcinoembryonic antigen levels were within the normal range. Other tests, including blood biochemistry, thyroid function, and sex hormones, showed no significant abnormalities. The remaining positive findings are presented in the Table 1 as shown below.

**Table 1:** Laboratory findings at admission.

Parameter	Measured value	Normal range	Interpretation
White blood cell count (WBC)	8.6 × 10^9^	3.5–9.5 × 10^9^/L	Normal
Lymphocyte percentage	33.3	20%–40%	Normal
Neutrophil percentage	56.6	50%–70%	Normal
Serum amyloid A (SAA)	139.71	0–10 mg/L	Elevated
Interleukin-6 (IL-6)	22.06	0–7 pg/mL	Elevated
Procalcitonin (PCT)	1.215	0–0.5 pg/mL	Elevated
25-Hydroxyvitamin D	25.6	30–100 ng/mL	Low

### Imaging findings

**Figure 1:**
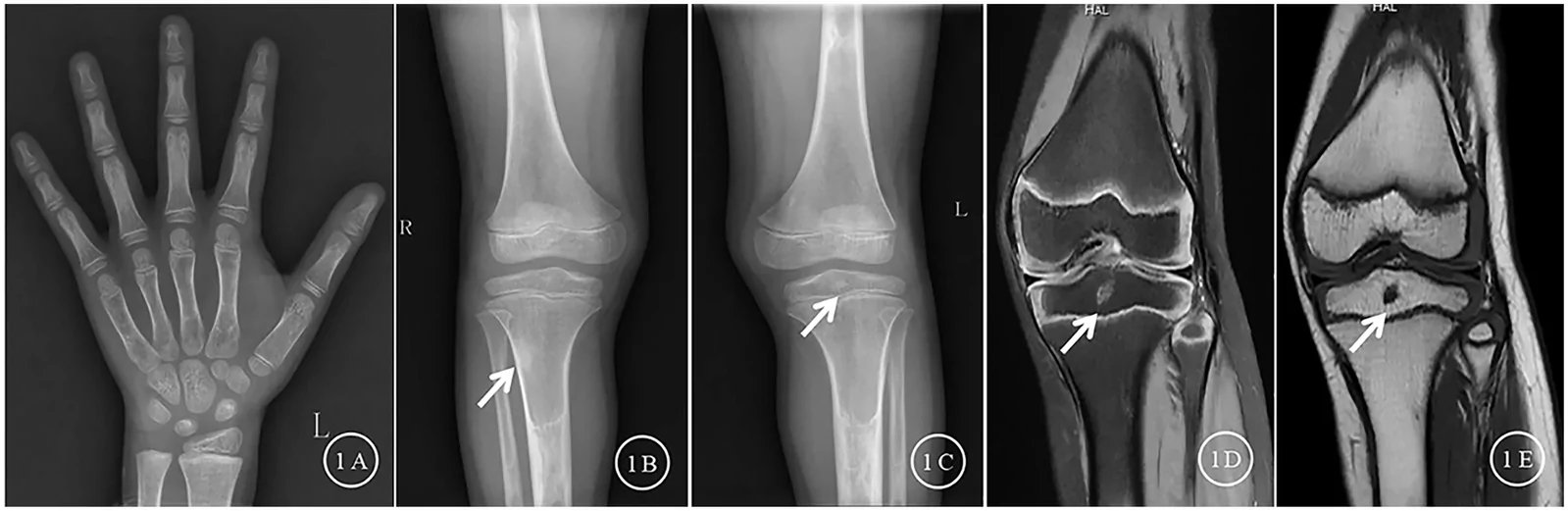
Pediatric ECD with epiphyseal involvement. **(A)** Bone age X-ray: chronological age 11 years 5 months, R-series bone age 8 years 3 months, and C-series bone age 8 years 7 months. **(B,C)** X-ray of both knees: patchy high-density shadows in the left tibial epiphysis and lateral edge of the right tibial metaphysis (white arrows). **(D,E)** MRI of the left knee showing: patchy hypointensity on T1WI and hyperintensity on T2WI in the left tibial epiphysis with clear margins (white arrows).

Bone age X-ray (Figure 1): (Figure 1A) Chronological age was 11 years 5 months; bone age by the R series was 8 years 3 months; and by the C series was 8 years 7 months. X-ray of both knees (Figures 1B,C): Patchy high-density shadows were seen in the left tibial epiphysis and the lateral edge of the upper end of the right tibia. MRI of the left knee (Figures 1D,E): The left tibial epiphysis showed a well-defined patchy hypointensity on T1WI and hyperintensity on T2WI.

**Figure 2:**
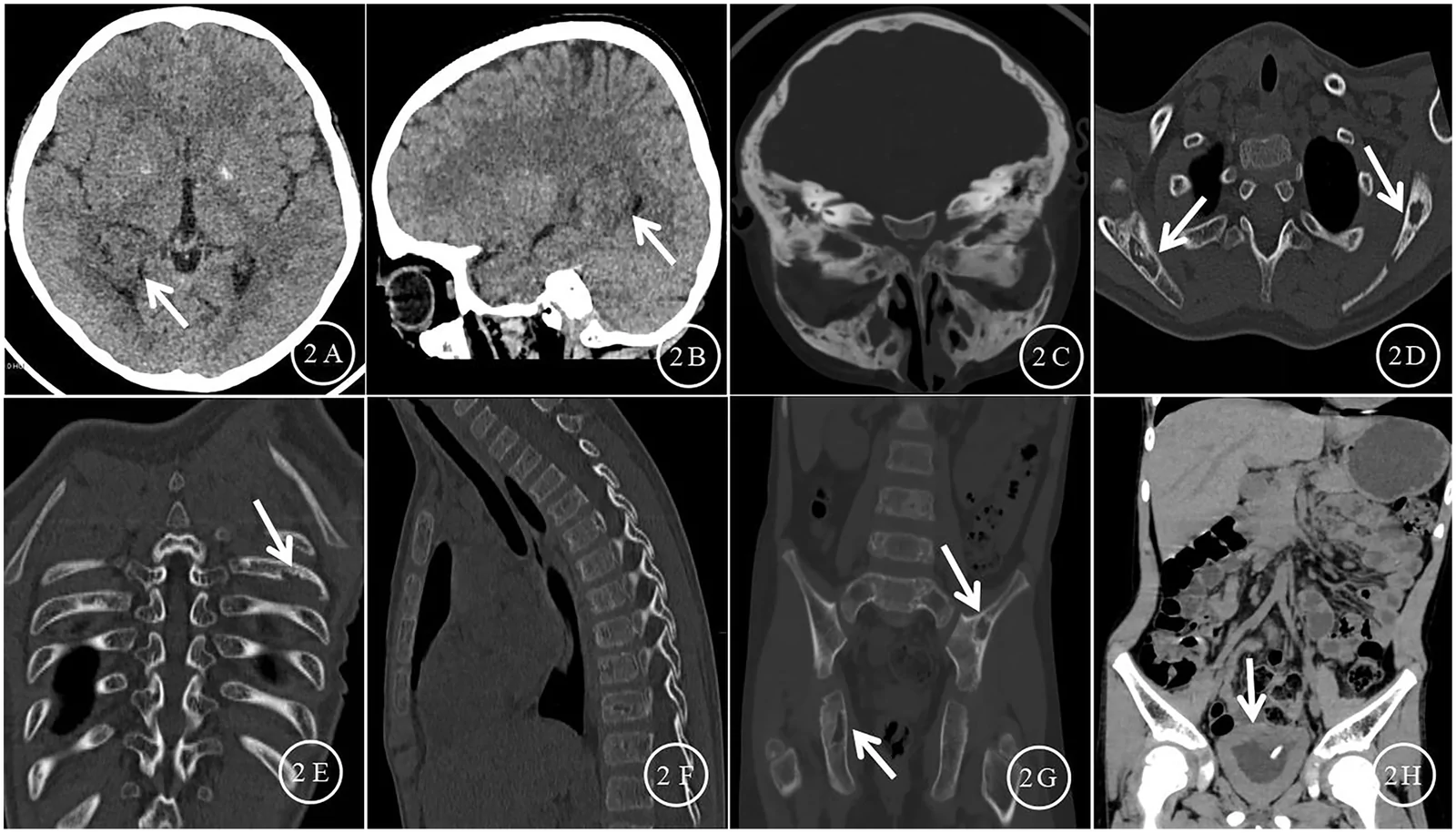
CT findings of ECD. **(A,B)** Axial and sagittal CT: a knotted, patchy, mixed-density lesion in the right lateral ventricle trigone (white arrow). **(C–G)** Extensive hyperdensity of the facial bones, skull base, and calvarium, with irregular contours; heterogeneous bone density of the scapulae, sternum, ribs, vertebrae, and pelvis (white arrows). **(H)** Diffuse bladder wall thickening (≈1.6 cm, white arrow).

Computed tomography (CT) of the skull, thorax, and abdomen (Figure 2): In the trigone of the right lateral ventricle, a knotted, patchy, mixed-density (isodense, slightly hyperdense, and hypodense) lesion was observed, with indistinct margins from the adjacent brain parenchyma at some levels. Extensive hyperdensity was observed in the maxillofacial bones, anterior and middle cranial fossae, and bilateral frontotemporo-occipital bones, some with irregular contours. The bilateral scapulae, sternum, several ribs, thoracolumbar vertebrae and pelvic bones showed heterogeneous bone density. The bladder wall was diffusely thickened (≈1.6 cm). The remainder of the abdominal examination was unremarkable.

**Figure 3:**
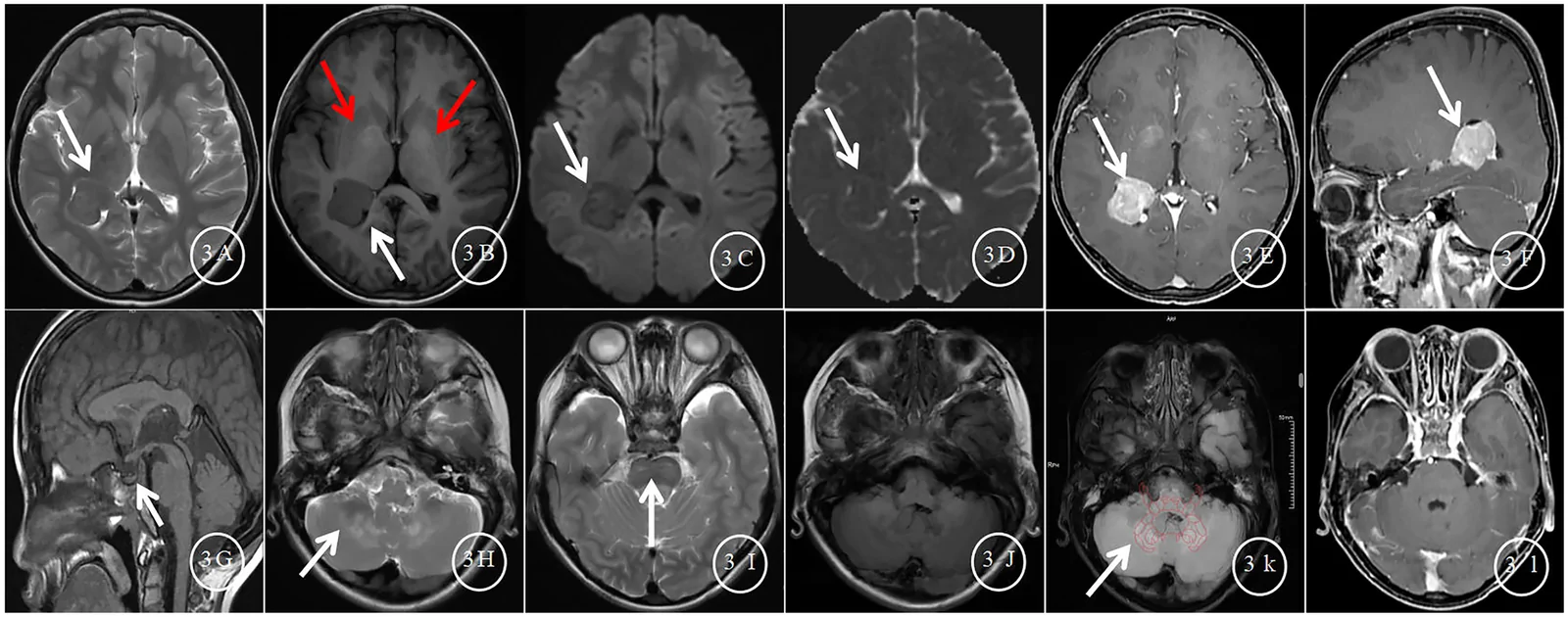
MRI findings of ECD. **(A,B)** Axial T1-weighted (T1W) and T2-weighted (T2W) images: a round lesion in the right lateral ventricle trigone showing slightly hypointense signal on T1WI and iso-to-slightly hyperintense signal on T2WI (white arrow); patchy hyperintense signals in the bilateral basal ganglia (red arrows). **(C,D)** Diffusion-weighted imaging (b = 1,000 s/mm^2^): no restricted diffusion (white arrow). **(E,F)** Contrast-enhanced T1WI: marked homogeneous enhancement of the ventricular lesion. **(G)** Sagittal T1-weighted non-contrast image: normal sella turcica with absence of the posterior pituitary bright spot (white arrow). **(H–K)** Axial T1WI and T2WI images: ill-defined lesions in the bilateral cerebellar hemispheres, pons and cerebral peduncles, appearing hypointense on T1W and hyperintense on T2WI/FLAIR, resembling a “crab” (K, white arrow). **(L)** No enhancement on post-contrast T1WI.

Cranial MRI (Figure 3): In the right lateral ventricle trigone, a round lesion with a slightly hypointense signal on T1WI and iso-to-slightly hyperintense signal on T2WI, well-defined margins, and no perilesional edema was observed. The lesion was irregularly shaped and contained small blood vessels. On contrast-enhanced T1WI, the lesion showed marked homogeneous enhancement, measuring approximately 2.7 cm × 2.8 cm × 2.6 cm. On T1-weighted images, patchy slightly hyperintense signals with blurred borders were observed in the bilateral basal ganglia. Patchy hypointensity on T1WI and hyperintensity on T2WI with ill-defined margins were observed in the bilateral cerebellar hemispheres, pons and bilateral cerebral peduncles of the midbrain; these lesions did not enhance. Sellar MRI: The sella turcica was not enlarged. The superior margin of the pituitary gland was straight, and the posterior pituitary bright spot was not observed. The pituitary stalk was not displaced from its normal position. No abnormal signals or enhancements were detected.

### Surgery and histopathology

After multidisciplinary consultation, resection of the ventricular mass was performed. Intraoperatively, the tumor was located in the right lateral ventricle trigone, appeared light yellow, and had a clear boundary from the ventricular walls. Postoperative pathological examination revealed a histiocytic proliferation lesion.

**Figure 4:**
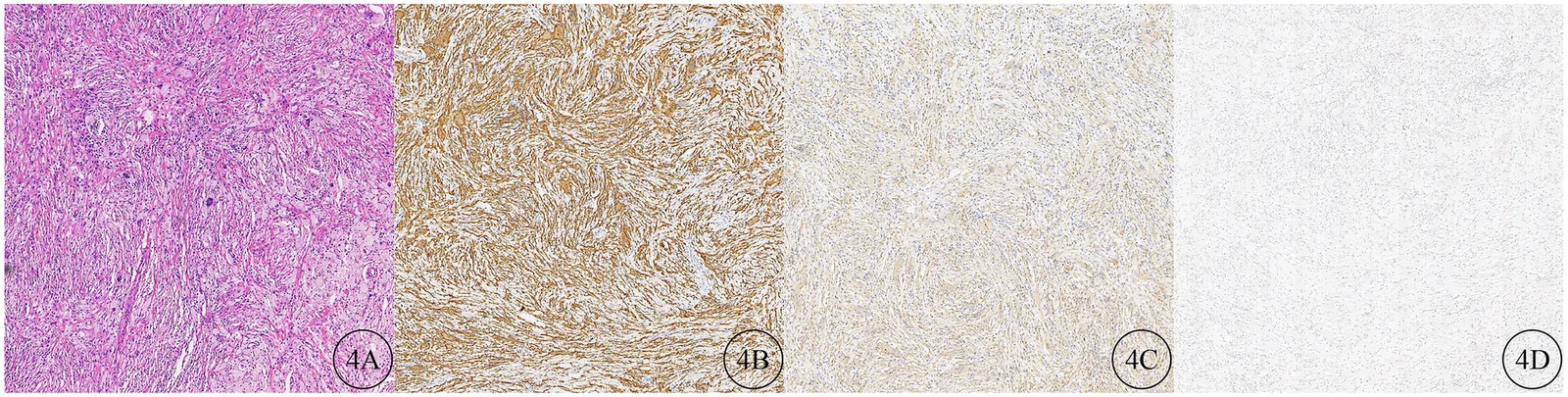
Histopathological features of ECD. **(A)** Histologic evaluation showed diffuse infiltration of the tissue by numerous lipid-laden foamy histiocytes, with scattered Touton multinucleated giant cells identified within the lesion. The lesional stroma demonstrated prominent collagenous fibrosis, associated with an infiltrate of chronic inflammatory cells consisting predominantly of lymphocytes and plasma cells (HE × 40). **(B,C)** Immunohistochemistry: foamy macrophages positive for CD163 and CD68 (×40). **(D)** Negative for CD1a (×40).

Pathological and Immunohistochemistry (Figure 4): Histologic evaluation (hematoxylin and eosin, HE) showed diffuse infiltration of the tissue by numerous lipid-laden foamy histiocytes, with scattered Touton multinucleated giant cells identified within the lesion. The lesional stroma demonstrated prominent collagenous fibrosis, associated with an infiltrate of chronic inflammatory cells consisting predominantly of lymphocytes and plasma cells.

Positive markers: CD68 (+), CD163 (+), S-100 (<5%), Ki-67 (<5%), and EMA (<5%). Negative markers: CD1a (–), ALK (–), GFAP (–), and PR (–). BRAF V600E mutation analysis: positive with a mutation rate of 6.40%. Pathological consultation confirmed the diagnosis of ECD.

### Treatment and follow-up

The management of our patient was guided by the 2020 International Consensus Recommendations for the Evaluation, Diagnosis, and Treatment of ECD (7). According to these guidelines, for patients with BRAF V600E-mutated ECD and clinically significant or life-threatening disease, targeted BRAF inhibitor therapy (vemurafenib or dabrafenib) is recommended as the first-line treatment. In July 2024, he was initiated dabrafenib treatment, interferon α-2b, and desmopressin acetate for diabetes insipidus. After two months of treatment, his clinical symptoms improved dramatically compared with his postoperative baseline (Table 2). And increasing studies have demonstrated that BRAF V600 kinase inhibitors (vemurafenib or dabrafenib) can cross the blood-brain barrier and exert sustained efficacy in patients with BRAF V600E-mutant ECD (8, 9). However, data on dabrafenib in pediatric ECD cases with CNS involvement are limited. The positive clinical outcomes achieved with dabrafenib therapy in our pediatric ECD case offer additional supporting evidence for its clinical application. However, it is important to acknowledge that the clinical improvement observed in this patient cannot be attributed solely to dabrafenib therapy. The patient underwent surgical resection of the ventricular mass, which likely contributed to symptomatic relief by alleviating mass effect. This is supported by the fact that the patient was able to walk independently at 4 months post-surgery, before any targeted therapy had been initiated. In ECD, surgical resection is generally not curative; its primary roles are to establish a tissue diagnosis through biopsy and to achieve debulking for relief of mass effect when clinically indicated (10). However, ECD is a systemic neoplastic disorder driven by activating MAPK pathway mutations, and sustained disease control requires effective systemic therapy. The subsequent marked improvement following initiation of dabrafenib, and the rapid deterioration after self-discontinuation, provides strong evidence that targeted therapy played a critical role in maintaining disease control. Therefore, in our case, we consider that the overall clinical improvement was likely attributable to the combined effect of surgical debulking and targeted therapy, with dabrafenib being essential for sustained disease control.

**Table 2:** Detailed medication regimen, dosing, and neurologic assessments over time.

Time point	Medication	Dosage/Strength	Frequency	Duration	Adherence	Neurologic examination findings
First hospitalization (July 27, 2024)	Dabrafenib	1/2 tablet (37.5 mg)/dose	Twice daily	2 months	Regular	Admission: Unsteady gait; right lower limb muscle strength grade IV, left lower limb grade III; knee reflexes and abdominal reflexes normally elicited; bilateral pathological signs (-). Discharge: Improved lower limb activity.
	Interferon α-2b	1.8 million units	3 times/week	2 months	Regular	
	Desmopressin acetate	1 tablet/dose	3 times/day	2 months	Regular	
	Prednisone	4 tablets/day × 1 day (Aug 24); tapered to 2 tablets/day × 3 days (Aug 25–27); 1 tablet/day × 3 days (Aug 28–30), then discontinued				
Second hospitalization (September 27, 2024)	Dabrafenib	1/2 tablet (37.5 mg)/dose	Twice daily	2 months	Intermittent	Admission: Able to walk independently but with abnormal gait; speech delay. Discharge: Able to walk independently with stable gait; speech delay.
	Interferon α-2b	Discontinued	-	-	-	
	Desmopressin acetate	1 tablet/dose	3 times/day	2 months	Regular	
Third hospitalization (November 25, 2024)	Dabrafenib	1 tablet (75 mg)/dose	Once daily	Ongoing	Intermittent	Admission: Stable gait with dragging, more pronounced on left side; speech delay. Discharge: Improved lower limb activity; stable mental status.
	Desmopressin acetate	1 tablet/dose	3 times/day	Ongoing	Regular	
Final follow-up (April 2026)	Dabrafenib	-	-	-	Treatment interruption	Unable to walk; significant speech delay.

Unfortunately, the follow-up period was 32 months. By April 2026, the patient had become non-ambulatory and exhibited speech delay (Table 3). This deterioration may be partly attributable to poor adherence to the prescribed medication regimen. Medication adherence was assessed through parental reporting during clinical interviews and follow-up telephone calls. At the final follow-up (April 2026), the parents reported that after the last hospitalization (November 25, 2024), the patient had completed one full bottle (120 tablets) of dabrafenib dispensed at discharge, and had not visited any medical institution to purchase a refill thereafter, resulting in complete treatment cessation. The exact date of discontinuation could not be specified by the parents. It has been demonstrated that even a brief cessation of dabrafenib treatment can result in rapid, life-threatening disease progression (11). For pediatric hematologists, this case strongly demonstrates that continuous administration of dabrafenib is essential for controlling disease in children with BRAF V600E-mutated ECD. Particular caution is advised when withdrawing dabrafenib in ECD patients with CNS involvement.

**Table 3:** Timeline of diagnosis, treatment, and follow-up in the present case.

Time points (T0 = day of surgery)	Key events and outcomes
T −48 months	Parents noticed that the patient's height had not increased and was significantly lower than that of peers of the same age and sex
T −24 months	Unsteady gait; inability to climb stairs or slopes independently
T −1 month	Painful micturition; increased nocturnal urine volume and frequency.
T −1 month to T0	Imaging findings: right lateral ventricular mass; symmetrical white matter lesions in the bilateral cerebellar hemispheres, pons, and bilateral cerebral peduncles; loss of the posterior pituitary bright spot; epiphyseal lesions of the knee joints; extensive osteosclerosis; diffuse bladder wall thickening
T0	The ventricular mass resection performed. Postoperative pathological examination revealed a histiocytic proliferative lesion. Immunohistochemistry: CD68 (+), CD163 (+), S-100 (<5%); Ki-67 (<5%); EMA (<5%), CD1a (–)
T +4 months	Patient could walk independently
T +11 months	Recurrence of unsteady gait (progressive); painful micturition. CT showed persistent diffuse bladder wall thickening
T +12 months	BRAF V600E mutation analysis: positive (mutation rate 6.40%). Pathological consultation confirmed the diagnosis of ECD. Treatment initiated: dabrafenib, interferon α-2b, and desmopressin acetate for diabetes insipidus
T +14 months	Clinical symptoms improved dramatically
T +15 months	Patient self-discontinued dabrafenib and interferon without medical advice; irregular treatment. Gait abnormality and polyuria recurred
T +32 months	Unable to walk; speech delay

## Discussion

ECD can involve multiple organs, and the classic triad comprises bone pain, central diabetes insipidus, and exophthalmos (3). Skeletal involvement occurs in 96% of patients, preferentially affecting the long bones (femur, tibia, and fibula) and occasionally the skull, mandible, ribs, and pelvis (4, 12–14), sparing the axial skeleton, as well as the hands and feet. Imaging typically shows symmetric osteosclerosis of the long bones, occasionally accompanied by lytic changes (3). Epiphyseal involvement is considered rare (15, 16); however, Dion et al. reported epiphyseal changes in 45% of their ECD cohort, but all of these were observed in adults (17). In the present case, X-ray of both knees showed patchy osteosclerosis of the tibial epiphyses, and MRI demonstrated well-defined areas of hypointensity on T1WI and hyperintensity on T2WI—the first description of MRI findings of epiphyseal involvement in Pediatric ECD. Unlike adult ECD, which predominantly affects the diaphysis and metaphysis of long bones. Our pediatric case demonstrates epiphyseal involvement on imaging, which may represent either direct infiltration by abnormal foamy histiocytes potentially favored by the high metabolic activity of the open physis during childhood, or secondary effects of endocrine dysregulation. Indeed, the patient's pituitary abnormalities (small pituitary gland and absent posterior pituitary bright spot) raise the possibility of growth hormone deficiency secondary to hypothalamic-pituitary axis involvement, which could also explain the growth retardation. In the absence of pathological confirmation of ECD within the epiphyses, and without IGF-1 and IGFBP-3 levels to evaluate the growth hormone axis, we are unable to definitively distinguish between these two mechanisms. We therefore present both as plausible hypotheses worthy of further investigation. Regardless of the exact mechanism, the epiphyseal lesions observed on imaging are clinically significant, as the physis is responsible for longitudinal bone growth, and its involvement by ECD poses a unique threat to pediatric patients. Therefore, recognizing atypical epiphyseal lesions in children with unexplained bone pain or gait abnormalities is crucial for early diagnosis and preserving future skeletal development. In addition, cranial, thoracic, and abdominal CT in our case revealed extensive osteosclerosis of the maxillofacial bones, the anterior and middle cranial fossae, and the bilateral frontotemporo-occipital bones, some with irregular contours. Furthermore, heterogeneous bone density, predominantly sclerotic, was observed in the bilateral scapulae, sternum, several ribs, thoracolumbar vertebrae, and pelvic bones. These axial skeletal manifestations constitute critically important imaging differential features in pediatric ECD.

ECD is a rare multisystem disorder with a highly variable clinical course (18). Central nervous system (CNS) involvement occurs in 25%–50% of patients and can affect the hypothalamic-pituitary axis, brain parenchyma, dura mater, spinal cord, and orbits (19). Notably, ECD can also present purely with neurological manifestations, as recently reported by Ramos et al., who described a case with isolated brainstem and cerebellar involvement (20). Dural involvement may present as diffuse thickening or meningioma-like masses, although it was not observed in our case. Hypothalamic-pituitary axis abnormalities are common; Drier et al. reported the loss of the normal T1WI hyperintense signal of the posterior pituitary lobe in 88% of affected patients (21). Our patient had a small pituitary gland and an absent posterior pituitary bright spot, which is consistent with these observations.

The ventricular mass in our patient exhibited marked homogeneous enhancement, initially raising the suspicion of ependymoma or meningioma. To the best of our knowledge, this is the third reported case of ECD presenting as a ventricular mass (4, 6). To provide a clearer overview of the three reported pediatric cases of ECD with cerebral ventricular system involvement, we have summarized their key clinical, radiological, pathological, and therapeutic features in Table 4 for side-by-side comparison (for abbreviation definitions, see Table 4 footnote a). Ma et al. described an 8-year-old child with polydipsia and polyuria who was found to have a lateral ventricular lesion (6). Compared with their case, our patient had a longer symptomatic period (2 years vs. 4 years of growth retardation prior to presentation). Additionally, bilateral basal ganglia and cerebellar white matter lesions with ill-defined margins and no contrast enhancement were observed, findings consistent with CNS parenchymal involvement. Parks et al. reported T2 white matter hyperintensities in 61.7% (21/34) of ECD patients, with only two showing gadolinium enhancement (22). Similar white matter changes were described by Zahergivar et al. (23) and Mustafa et al. (24). Mustafa et al. introduced the “crab sign”—a novel radiological sign in CNS ECD that resembles a crab (24). This sign is characterized by central cavitation within the dentate nuclei at the mid-pons level on FLAIR images, appearing hypointense on T1-weighted and hyperintense on T2/FLAIR sequences. Recognizing this sign may facilitate early diagnosis and potentially prevent disease progression along the neuraxis. Consistent with these findings, we observed this sign in our case (Figure 3K). After 32 months of follow-up, the patient's condition progressed from an unsteady gait to an inability to walk and speech delay, possibly reflecting the spread of the “crab-like” lesions along the neuraxis.

**Table 4:** Comparison of reported pediatric cases of ECD with cerebral ventricular system involvement.

Feature	Khalak S (2026) (4)	Ma et al. (2022) (6)	Present case
Age at presentation	2 years	8 years	11 years
Sex	Male	Male	Male
Initial symptoms	Polydipsia, polyuria (central diabetes insipidus)	Polydipsia, polyuria (4 years), limited left eye movement	Gait disturbance, growth retardation (4 years), left knee pain, dysuria, nocturia
Time to diagnosis	∼2 years (from age 2 to 4)	Not specified (4 years of symptoms before diagnosis)	∼2 years (from symptom onset to diagnosis)
Ventricular lesion	Multiple intraventricular nodular lesions	Large mass in lateral ventricle	Round mass in right lateral ventricle trigone (2.7 × 2.8 × 2.6 cm)
Other CNS^a^ involvement	Orbital/retrobulbar infiltration, parasellar lesions, venous sinus involvement	Limited left eye movement	Bilateral cerebellar white matter lesions (“crab sign”), bilateral basal ganglia lesions, absent posterior pituitary bright spot
Skeletal involvement	Paravertebral masses (Th4–Th6)	Not reported	Extensive osteosclerosis (skull, facial bones, scapulae, ribs, vertebrae, pelvis); tibial epiphyseal lesions^b^
Urinary involvement	Bilateral obstructive uropathy, megaureter, hydronephrosis, retroperitoneal masses	Not reported	Diffuse bladder wall thickening (1.6 cm)^b^
Endocrine involvement	Central diabetes insipidus	Central diabetes insipidus (polydipsia/polyuria)	Central diabetes insipidus (absent posterior pituitary bright spot); growth retardation
Immunohistochemistry^b^	CD68+, CD14+, CD1a−, Factor XIIIa−	CD68+, CD163+, CD1a−, langerin−, S100 partial+	CD68+, CD163+, CD1a−, S100< 5%, Ki-67< 5%
BRAF V600E mutation^b^	Positive (tissue-based molecular analysis)	Positive (IHC and RT-qPCR)	Positive (6.40%, dPCR)
Treatment	Dabrafenib (5.25 mg/kg/day)	Dexamethasone+vincristine, then dabrafenib	Dabrafenib, interferon α-2b, desmopressin
Treatment response	Clinical and radiological response within 3 months; sustained at 6–12 months	Neurological symptoms improved	Initial improvement; deterioration after self-discontinuation; non-ambulatory with speech delay at 32 months
Follow-up duration	12 months	Outpatient follow-up (duration not specified)	32 months

^a^ECD, Erdheim-Chester disease; CNS, central nervous system; IHC, immunohistochemistry; RT-qPCR, reverse transcription-quantitative polymerase chain reaction.

^b^All three cases were male and harbored BRAF V600E mutations, with a consistent immunohistochemical profile of CD68+/CD1a−. However, the present case is distinguished by epiphyseal involvement and diffuse bladder wall thickening, which have not been previously reported in pediatric ECD.

Regarding urinary tract involvement, ECD typically affects the kidneys, ureters, and renal arteries, with the classic “hairy kidney” sign representing perirenal soft tissue infiltration (12, 25–27). In a large series of 47 ECD patients (28), Yelfimov et al. reported urologic involvement in 79% of cases, including retroperitoneal infiltration (60%), worsening lower urinary tract symptoms from diabetes insipidus (45%), hydronephrosis (21%), and chronic kidney disease (38%). Our patient experienced painful micturition and increased nocturnal frequency. CT revealed irregular thickening of the bladder wall (1.6 cm). One year later, repeat CT at another hospital still showed diffuse bladder wall thickening (1.5 cm). Cystoscopic biopsy was recommended to establish a definitive etiology; however, the patient's parents declined the procedure, and their decision was fully respected. Although the patient's blood and urine leukocyte counts were within normal limits and isolated cystitis remained a diagnostic consideration, we deemed it unlikely to account for thickening that had persisted for an entire year. A review of the literature revealed that bladder wall thickening has not been previously described as a manifestation of ECD in either the pediatric or adult literature. On the basis of these observations, we cautiously hypothesize that bladder wall thickening ≥1.5 cm might represent a novel imaging sign of ECD involving the urinary system. Direct histological confirmation is lacking, and this observation should be considered as preliminary.

### Diagnostic workup and differential diagnosis of ECD

The diagnosis of ECD is often challenging due to its nonspecific inflammatory and fibrotic histopathological appearances, as well as its insidious onset, which frequently results in diagnostic errors and delays. A high degree of suspicion and a systematic, multidisciplinary approach integrating clinical, radiological, histopathological, and molecular findings are therefore required for accurate diagnosis.

Clinical and radiological suspicion: The diagnostic process begins with recognition of characteristic clinical and imaging features. In our case, the constellation of progressive gait disturbance, growth retardation, left knee pain, dysuria and nocturia, combined with imaging findings of a right lateral ventricular mass, bilateral cerebellar white matter lesions exhibiting the “crab sign”, patchy tibial epiphyseal osteosclerosis, and diffuse bladder wall thickening, raised strong suspicion for a systemic histiocytic disorder.

Histopathological and immunohistochemical confirmation: The cornerstone of ECD diagnosis is tissue biopsy with histopathological and immunohistochemical analysis. ECD is characterized by infiltration of foamy histiocytes that are CD68-positive (+) and CD1a-negative (−). The immunohistochemical profile is essential for distinguishing ECD from other histiocytic disorders, including Langerhans cell histiocytosis (LCH), Rosai-Dorfman disease (RDD), juvenile xanthogranuloma (JXG), and reactive histiocytic proliferations (Table 5).

**Table 5:** Summarizes the key differentiating features of these entities.

Feature	ECD	LCH	RDD	JXG
Immunohistochemistry	CD68+, CD1a−, S100−/focal+	CD68+, CD1a+, S100+, langerin+	CD68+, CD1a−, S100+	CD68+, CD1a−, S100−, Factor XIIIa+
Morphology	Foamy histiocytes in fibrotic background; multinucleated giant cells	Grooved nuclei; eosinophilic infiltrate	Large foamy histiocytes; emperipolesis	Touton giant cells; xanthogranulomatous morphology
Key clinical features	Long bone osteosclerosis; diabetes insipidus; perirenal fibrosis; CNS involvement	Osteolytic lesions; lung involvement; skin rash	Massive lymphadenopathy; extranodal involvement	Skin lesions; usually self-limited in children
BRAF V600E mutation	∼54% (adults); ∼60% (pediatric)	∼38%–57%	Absent	Rare (CNS JXG subset)

Molecular confirmation: Molecular analysis for MAPK pathway mutations, particularly BRAF V600E, is now an integral part of the diagnostic workup. The critical role of molecular analysis was underscored by Haroche et al., who, in a seminal multicenter study, detected BRAF V600E mutations in over half (54%) of the ECD cohort, a frequency strikingly similar to that observed in Langerhans cell histiocytosis (29). This finding not only highlighted a shared pathogenetic pathway between these two histiocytic disorders but also established BRAF mutation testing as a standard of care, guiding the use of targeted therapies. In our pediatric patient, the detection of the BRAF V600E mutation (6.40%) by digital PCR was pivotal, providing definitive diagnostic confirmation and directly informing the decision to initiate dabrafenib therapy.

Integrated diagnosis: The final diagnosis of ECD requires integration of all components: compatible clinical presentation, characteristic imaging findings, CD68+/CD1a− immunohistochemical profile, and, when present, confirmation of an activating MAPK pathway mutation. Reactive histiocytic proliferations, in particular, are polyclonal and do not harbor BRAF or other MAPK pathway mutations, distinguishing them from neoplastic histiocytoses such as ECD.

This case report has several notable strengths. First, the use of comprehensive multimodal imaging—including radiography, CT, and MRI—enabled detailed characterization of both intracranial and extracranial disease manifestations, which is particularly valuable given the paucity of imaging data in pediatric ECD. The systematic documentation of imaging findings across multiple body regions provided a robust foundation for diagnosis and facilitated the identification of novel radiological signs, including epiphyseal involvement and diffuse bladder wall thickening, which have not been previously described in the pediatric ECD literature. Specifically, skeletal assessment was performed using conventional radiography, which demonstrated bilateral cortical osteosclerosis of the metaphysis and diaphysis of the long bones, a characteristic finding of ECD. MRI of the left knee further delineated the extent of medullary involvement and clearly depicted the epiphyseal lesions with well-defined hypointensity on T1WI and hyperintensity on T2WI. For visceral and intracranial evaluation, contrast-enhanced CT and MRI were systematically employed, as these modalities are considered the most useful for detecting extraskeletal involvement. Cranial MRI with contrast was particularly critical in this case, as it not only characterized the right lateral ventricular mass but also revealed bilateral cerebellar white matter lesions exhibiting the “crab sign”, bilateral basal ganglia lesions, and absence of the posterior pituitary bright spot—findings that are consistent with the diverse neuroimaging spectrum of CNS ECD. CT of the chest, abdomen, and pelvis further documented extensive osteosclerosis of the axial skeleton and diffuse bladder wall thickening, expanding the known imaging phenotype of pediatric ECD. Second, the 32-month follow-up period represents one of the longest documented clinical trajectories for a pediatric ECD patient with cerebral ventricular system involvement, allowing valuable insights into the natural history of the disease and the clinical consequences of treatment interruption. Third, the integration of histopathological, immunohistochemical, and molecular data, with confirmation of the BRAF V600E mutation—provided definitive diagnostic confirmation and guided targeted therapy, underscoring the importance of a multidisciplinary diagnostic approach in this rare disease. Finally, our detailed documentation of the patient's clinical course, including the deterioration following self-discontinuation of dabrafenib, offers practical clinical lessons for pediatric hematologists managing similar cases.

### Limitations

This study had several limitations. First, molecular analysis was restricted to the BRAF V600E mutation; broader next-generation sequencing (NGS) was not performed to exclude other MAPK pathway alterations. Second, whole-body ^18^F-FDG PET/CT, the gold standard for staging histiocytic disorders, was unavailable at our institution, limiting the comprehensive assessment of the systemic disease burden and treatment response. Third, the lack of histological confirmation of bladder wall involvement warrants cautious interpretation of the proposed imaging signs. Fourth, because the patient underwent both surgical resection and targeted therapy, the relative contribution of each intervention to the observed clinical improvement cannot be definitively quantified. However, the temporal sequence, initial improvement after surgery alone, followed by sustained control with dabrafenib, and rapid deterioration upon discontinuation, strongly supports the essential role of continued targeted therapy in maintaining disease control. Fifth, medication adherence was assessed through parental reporting rather than objective measures such as pill counts or pharmacy refill records. While this may introduce recall bias, the close temporal relationship between reported treatment discontinuation and clinical deterioration supports the validity of this observation. Nonetheless, our systematic multimodal imaging documentation provides a valuable diagnostic reference for this rare disease.

## Conclusion

Pediatric ECD is an extremely rare multisystem disorder with diverse clinical manifestations and a complex disease course. The most important clinical message of this case is that continuous dabrafenib administration is essential for disease control in children with BRAF V600E-mutated ECD, particularly those with CNS involvement. Even a brief interruption of therapy can lead to rapid and devastating disease progression, as demonstrated by the marked deterioration observed in our patient following self-discontinuation. Pediatric hematologists should exercise extreme caution when considering any withdrawal of dabrafenib in this vulnerable population. Furthermore, this case expands the imaging phenotype of pediatric ECD by providing the first MRI description of epiphyseal involvement and proposing persistent bladder wall thickening (≥1.5 cm) as a potential novel imaging sign of urinary tract involvement, though histological verification is needed. The “crab sign” may facilitate early diagnosis of CNS involvement. Collectively, a multidisciplinary approach integrating imaging, histopathology, and immunohistochemistry is essential for accurate diagnosis, targeted therapy management, and avoidance of misdiagnosis in pediatric ECD.

## Patient perspective

The patient's parents shared the following perspective: We live in a remote rural area. Our son experienced difficulty walking and failure to grow for more than two years, and we were very worried. After visiting many hospitals, we finally received a definitive diagnosis at Gejiu People's Hospital, where he underwent resection of the brain tumor. When we learned that our son had such a rare disease, we felt scared and helpless. The doctors explained everything to us very clearly and, concerned that we might not have access to adequate treatment, proactively referred us to a higher-level hospital for further management. After starting the medication, we saw our son improve—he was able to walk again, and for the first time in years, we felt hopeful. However, when he self-discontinued the medication due to side effects and the burden of regular hospital visits, his condition deteriorated rapidly. Watching him lose the ability to walk again and struggle to speak has been heartbreaking. We hope that by sharing our experience, other families and doctors can learn from our lesson and avoid similar setbacks.

## Data Availability

The datasets presented in this article are not readily available because of ethical and privacy restrictions. Requests to access the datasets should be directed to the corresponding author.
